# Genome-Wide Identification, Characterization, and Expression Analysis of the NAC Transcription Factor Family in Sweet Cherry (*Prunus avium* L.)

**DOI:** 10.3390/plants14081201

**Published:** 2025-04-12

**Authors:** Feng An, Xin Yin, Kaibire Jueraiti, Yuanyuan Yang, Zhuoyang Yan, Jie Li, Dongqian Shan

**Affiliations:** College of Horticulture, Northwest A & F University, Yangling 712100, China; xiaodai_an@nwafu.edu.cn (F.A.); yinxin2022@nwafu.edu.cn (X.Y.); kaibire@163.com (K.J.); 18093135329@163.com (Y.Y.); a13603592442@163.com (Z.Y.); xlydmwt028@163.com (J.L.)

**Keywords:** sweet cherry, *NAC* gene family, phylogenetic analysis, gene expression

## Abstract

The NAC (NAM, ATAF1/2, and CUC2) family is one of the largest plant-specific transcription factor families, playing a crucial role in adaptation to abiotic stresses. However, the *NAC* gene family in sweet cherry (*Prunus avium* L.) remains poorly understood. In this study, we identified 130 *NAC* genes (*PaNAC*) from the sweet cherry genome, which were unevenly distributed across eight chromosomes. Phylogenetic analysis classified the PaNACs into 21 distinct groups, including 2 sweet cherry-specific groups. Comparative analysis revealed significant variations in gene proportions, exon–intron structures, and motif compositions among different groups. Furthermore, cis-element analysis suggested the potential roles of *PaNACs* in regulating plant growth, development, hormone signaling, and stress responses. Transcriptomic data revealed tissue-specific expression patterns for several *PaNAC* genes. qRT-PCR further confirmed that eight selected *PaNACs* were responsive to various abiotic stresses in Gisela 6, a widely used hybrid rootstock in sweet cherry production that shares high sequence similarity in *NAC* genes with *P. avium*. These findings provide valuable insights for future research on the functional characteristics of the *PaNAC* genes in the growth, development, and responses to abiotic stress in sweet cherry.

## 1. Introduction

Transcription factors (TFs) are proteins that bind to specific cis-acting elements in promoter regions, playing a crucial role in regulating various biological processes in plants [1,2]. NAC proteins represent one of the largest families of plant-specific transcription factors (TFs) [3]. The term “NAC” is derived from three genes that encode the conserved NAC domain—NAM (no apical meristem), ATAF1/2 (Arabidopsis thaliana Activation Factor 1/2), and CUC2 (cup-shaped cotyledon 2)—all of which possess a similar DNA-binding domain [4,5]. Most NAC proteins contain a highly conserved DNA-binding domain at the N-terminal region, with a central domain that is essential for transcriptional activation [6,7]. The C-terminal sequences, however, exhibit considerable divergence, determining the specificity of transcriptional activation [8,9]. The NAC domain comprises five subdomains (A to E) at the N-terminal, usually consisting of approximately 150 amino acid residues, which play a role in DNA binding, dimerization, and subcellular localization [10].

Since the identification of *NAC* genes, numerous studies have shown that NAC transcription factors are crucial for plant growth and development, including seed development [11,12], shoot tip meristem formation [13], lateral root formation [14], fiber development [15], floral morphogenesis [16], secondary cell wall synthesis [17], fruit ripening [18], and senescence [19,20]. Furthermore, *NAC* genes serve as essential regulators in plant responses to both abiotic and biotic stresses [21]. In *Solanum lycopersicum*, the overexpression of the NAC transcription factor *JUNGBRUNNEN1* improves drought tolerance [22]. Similarly, rice plants overexpressing *NAC1/ONAC022/ONAC045* exhibit enhanced drought resistance [23,24]. The overexpression of *OsNAC5* enhances cold tolerance in transgenic rice [25], while the overexpression of *ThNAC4* increases salt tolerance in *Tamarix* [26]. In *Prunus persica*, the transcription factor *PpNAC56* confers heat resistance when expressed in transgenic tomatoes [27]. Furthermore, *NAC028* and *OsNAC101* play positive roles in rice resistance to *Rhizoctonia solani* and *Fusarium fujikuroi*, respectively [28,29].

Sweet cherry (*Prunus avium* L.), a member of the Rosaceae family, is one of the most economically valuable deciduous fruit trees. Environmental changes significantly impact the growth of sweet cherry, particularly under abiotic stresses like salinity and drought [30]. Therefore, improving the stress tolerance of sweet cherry through molecular breeding is crucial. With the advent of high-throughput sequencing technology, an increasing number of plant species have had their genomes sequenced. As a result, the identification research of the *NAC* gene family is also increasing in plants, indicating a tendency of rapid expansion. Identification and expression studies of related gene families have been conducted in Arabidopsis [7], rice [31], wheat [32], apple [33], grapevines [34], and tomato [35]. However, no systematic study of the *NAC* family has been conducted in sweet cherry. Because *NAC* genes play key roles in many developmental processes and responses to abiotic stress, it is crucial to comprehensively study the *NAC* gene family in sweet cherry.

In this study, we conducted a genome-wide search to identify *NAC* family genes (*PaNACs*) in sweet cherry. Subsequently, we systematically analyzed the phylogenetic relationships, structural characteristics, chromosomal locations, orthologous gene pairs, and cis-elements of these *PaNAC* genes. Finally, based on RNA-seq data, we investigated the expression patterns of these genes across various tissues, and eight candidate genes were further validated through qPCR to assess their expression changes under abiotic stress. These findings provide a foundation for further understanding the role of *NAC* genes in the growth and development of sweet cherry, as well as its response to environmental stresses.

## 2. Results

### 2.1. Identification of NAC Genes in Sweet Cherry

In this study, 130 *NAC* genes were identified in the sweet cherry genome using a BLASTP-HMMER search strategy (see Methods). The *NAC* genes were named *PaNAC001* to *PaNAC130* based on their chromosomal locations (Table S1). We provided basic information for 130 *NAC* genes in sweet cherry, including the amino acid composition (AAs), molecular weight (MW), isoelectric point (pI), instability index, aliphatic index, and grand average of hydropathy (GRAVY) (Table S1). The lengths of the PaNAC protein sequences ranged from 152 (PaNAC123) to 755 (PaNAC086) amino acids, with an average length of 367 amino acids. The relative molecular weights (MWs) ranged from 17.67 kDa (PaNAC123) to 84.35 kDa (PaNAC086), with an average of 41.54 kDa. The pIs ranged from 4.33 (PaNAC072) to 9.95 (PaNAC109), with 97 members showing pI ≤ 7, and 33 members showing pI > 7, suggesting that most PaNAC proteins are weakly acidic. Subcellular localization analysis suggested that most NAC transcription factors are localized in the nucleus (Table S1). Furthermore, the chromosomal mapping of *PaNAC* genes revealed a non-random distribution across eight chromosomes in sweet cherry, highlighting the diversification and complexity of the NAC family (Figure S1). Chromosome 2 contained the most *NAC* genes (28), while chromosomes 3 and 5 contained the fewest (9). The distribution of *NAC* genes also showed clustering on certain chromosomes, such as *PaNAC025*-*PaNAC045* on chromosome 2 (Figure S1).

### 2.2. Phylogenetic Analysis of the NAC Proteins

**Figure 1:**
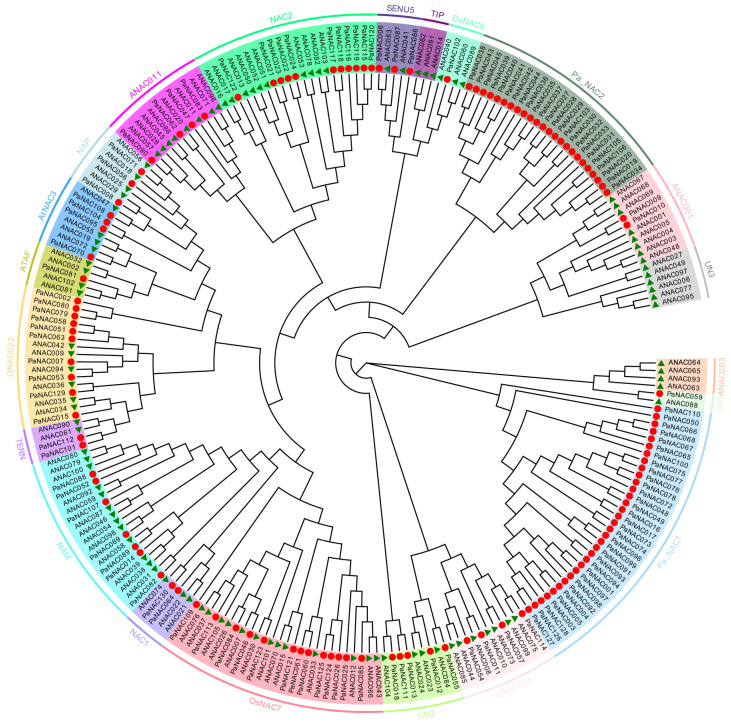
NJ phylogenetic tree constructed using NAC proteins from *A. thaliana* and *P. avium*. Different colors indicate distinct subfamilies. Circles represent *P. avium* PaNAC proteins, while triangles represent *A. thaliana* NAC proteins.

We constructed an unrooted phylogenetic tree using the amino acid sequences of PaNAC and AtNAC proteins to examine their evolutionary relationship (Figure 1). Based on the homology of NAC proteins in Arabidopsis, 130 protein sequences of sweet cherry were grouped into 21 groups, including 2 sweet cherry-specific groups named *Pa_NAC1* and *Pa_NAC2* (Figure 1). Each group contained different members. Pa_NAC1 was the largest, consisting of 33 TFs, followed by OsNAC7 with 26 TFs and Pa_NAC2 with 24 TFs. Except for *TIF*, *ANAC063*, *Pa_NAC1*, *Pa_NAC2*, and unclassified 3 (*UN3*), other groups contain AtNAC and PaNAC proteins, which indicates that *TIP* and *ANAC063* group loss occurred in the *NAC* gene family after sweet cherry and Arabidopsis differentiation.

### 2.3. Conserved Motifs and Gene Structure Analysis of PaNAC Genes

**Figure 2:**
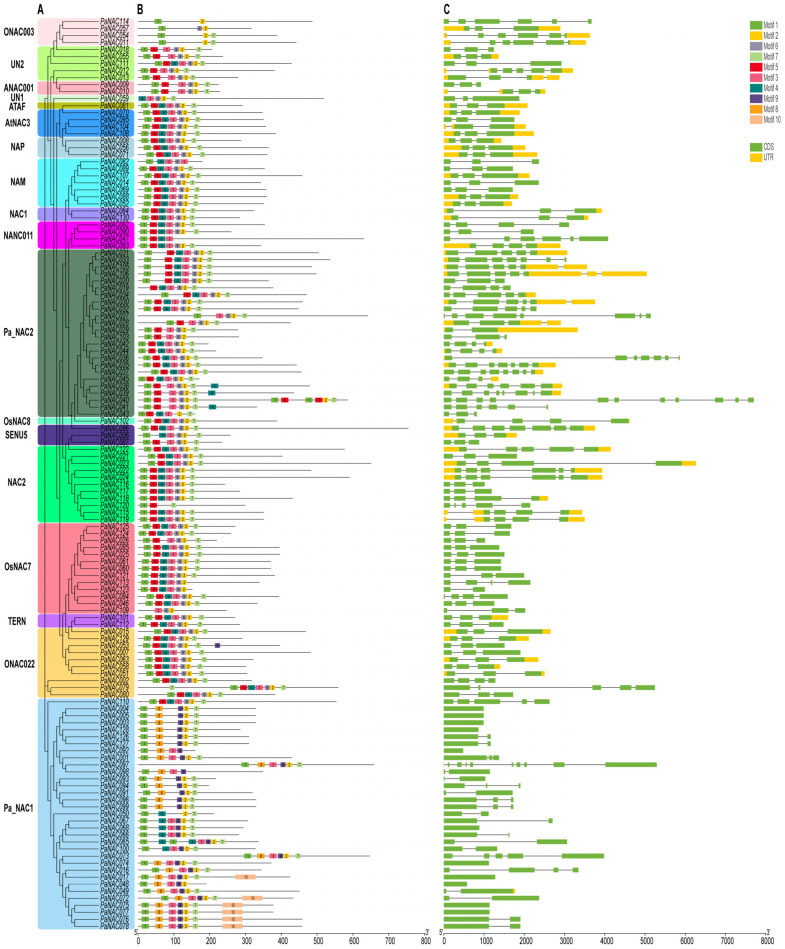
Phylogenetic tree, motifs, and gene structures of 130 PaNAC TFs. (**A**) The phylogenetic tree was constructed using the Neighbor-Joining (NJ) method with 1000 bootstrap iterations. (**B**) Positions of 10 motifs within the proteins are shown, with different colors representing distinct motifs. (**C**) Gene structures, with exons and untranslated regions (UTRs) indicated by green and yellow boxes, respectively, and introns represented by black lines. The ruler at the bottom shows the length scale for the gene features.

To gain further insights into the evolution of the *NAC* family in sweet cherry, we analyzed the gene structure and conserved motifs. Gene structure analysis revealed that 9 of the 130 *PaNAC* genes lacked introns, while the remaining genes contained at least 1 intron. The number of exons in the sweet cherry *NAC* genes ranged from 1 to 11 (Figure 2C). Building on the gene structure analysis, we next explored the conserved motifs in PaNAC proteins to further understand their structural diversity and potential functional implications. We analyzed the amino acid motifs of 130 PaNAC proteins using the MEME online tool (Figure 2B). The majority of *PaNAC* genes grouped into the same clade, exhibiting similar motif arrangements at corresponding positions, which suggests they may have similar biological functions. Most of the predicted motifs were concentrated in the N-terminus, which exhibited higher conservation. Motifs 1 through 6 were commonly found across most PaNAC proteins. Additionally, most PaNAC proteins contained at least 6 to 7 conserved motifs, while PaNAC120, PaNAC011, PaNAC054, and PaNAC109 contained only 3 each (Figure 2B).

### 2.4. Synteny Analysis of PaNAC Genes

**Figure 3:**
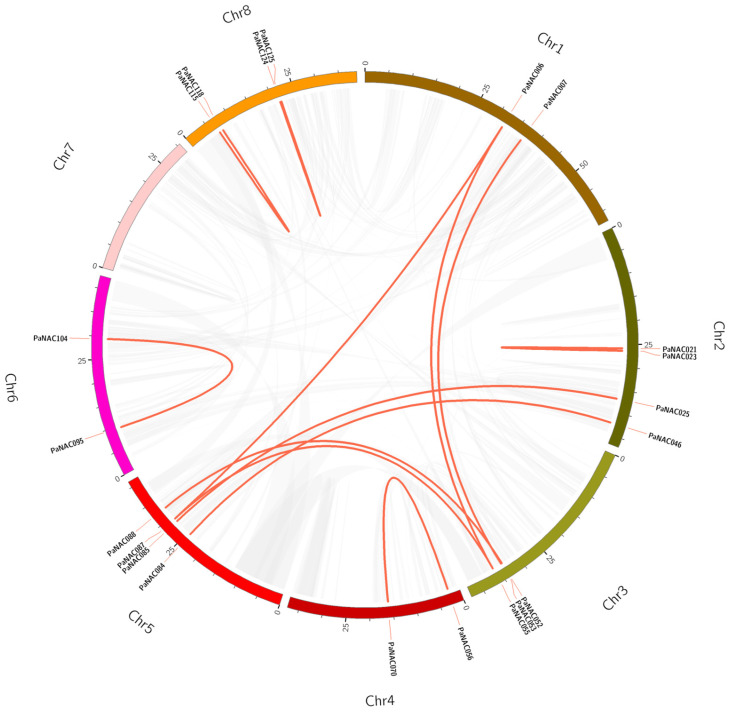
Collinearity analysis of the *P. avium NAC* gene family. Gray lines denote all synteny blocks in the sweet cherry genome; red lines denote the *PaNAC*-duplicated gene pairs.

To detect duplication events in *PaNAC* genes, synteny analysis was conducted using MCScanX v1.0.0 software. The results revealed segmental duplications in 12 *PaNAC* gene pairs and tandem duplications in 13 *PaNAC* gene pairs, most of which were located on chromosomes Chr1, Chr3, and Chr5 (Figure 3 and Table S2). Furthermore, we analyzed the evolutionary pressure between genes involved in segmental and tandem duplications (Table S2). The *Ka/Ks* values of all homologous *PaNAC* gene pairs were <1, indicating negative selection. This finding indicates that purifying selection likely contributed significantly to the evolution of *NAC* genes in sweet cherry.

**Figure 4:**
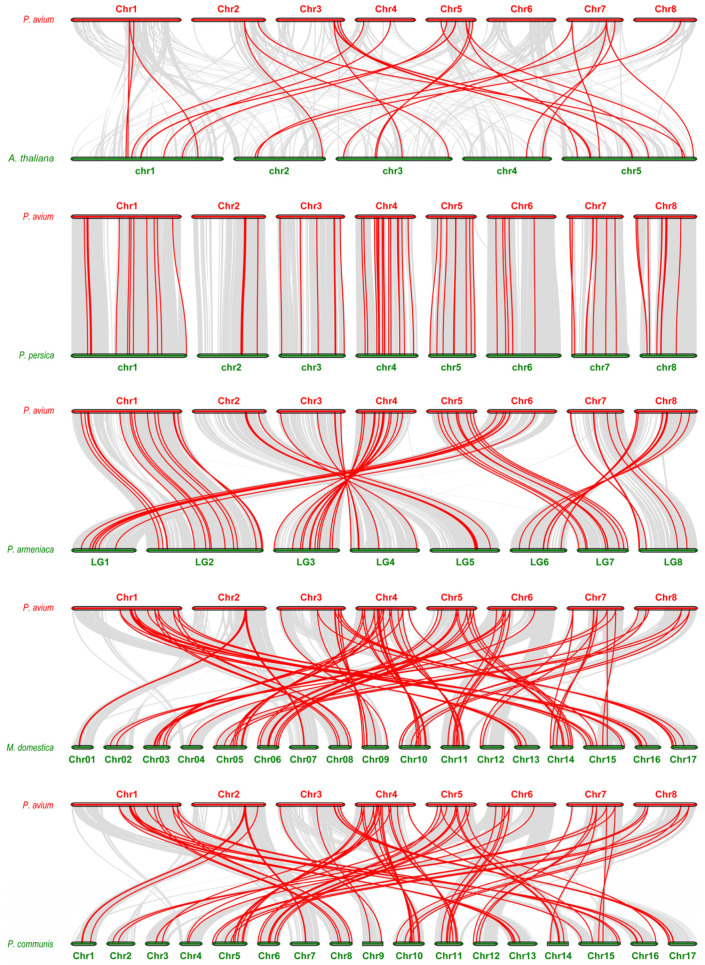
Synteny analysis of *NAC* genes in *P. avium* compared to *A. thaliana*, *P. persica*, *P. armeniaca*, *M. domestica*, and *P. communis*. Gray lines represent collinear blocks between *P. avium* and the other species, while colored lines highlight the collinear *NAC* gene pairs.

To further infer the origin and phylogenetic relationships of *NAC* genes, comparative collinearity analysis between *P. avium* and five representative species was conducted (Figure 4). The results reveal significant chromosomal conservation between *P. avium* and species such as *P. persica*, *P. armeniaca*, and *M. domestica*, particularly in chromosomal regions 1, 3, and 5. Further analysis suggests that during the genomic evolution of these species, syntenic regions likely represent conserved gene inheritance, while non-syntenic regions reflect species-specific gene variations.

### 2.5. Cis-Element Analysis of the PaNAC Genes

Numerous cis-elements were identified in the *PaNAC* gene promoter regions, including those involved in hormone signaling, such as abscisic acid (ABA), gibberellin (GA), methyl jasmonate (Me-JA), and auxin (Figure S2). The hormone signaling regions include ABRE, GARE-motif, CGTCA-motif, and TGA-box. Some promoters contained multiple abiotic stress response elements, such as LTR, MBS, and ERE, indicating that *NAC* genes play important roles in regulating plant abiotic stress responses. Additionally, some promoters contained several biotic stress response regions, particularly in the *PaNAC001* and *PaNAC098* genes (Figure S2).

### 2.6. Expression Profile of PaNACs in Different Tissues

**Figure 5:**
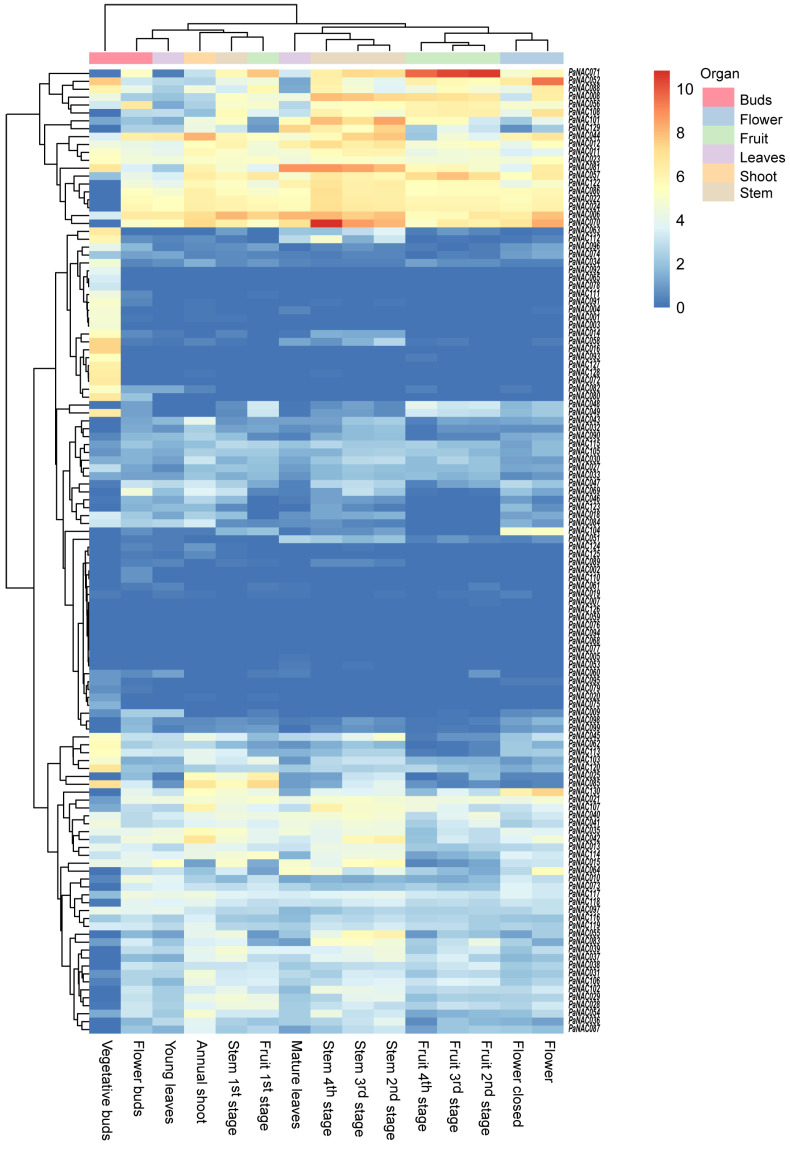
Expression patterns of the *PaNAC* gene family across different developmental stages and plant tissues.

To investigate the expression profiles of the 130 *PaNAC* genes in various tissues and developmental stages of sweet cherry, we utilized publicly available RNA-seq data for analysis. Seven *PaNAC* genes (*PaNAC026*, *PaNAC050*, *PaNAC066*, *PaNAC067*, *PaNAC100*, and *PaNAC109*) showed no expression across all tissues and developmental stages. After excluding these genes, a heatmap was created to visualize the expression levels of the remaining *PaNACs* using their Log (TPM + 1) values (Figure 5). Overall, the expression levels exhibit distinct tissue- and stage-specific variations. Notably, some *PaNACs* are highly expressed in specific tissues, while others show minimal or no expression at certain stages. *PaNAC120* and *PaNAC085* are significantly upregulated in vegetative buds, suggesting a potential role in bud dormancy and early development. Additionally, *PaNAC048* and *PaNAC049* exhibit higher expression during the fruit ripening, indicating their involvement in fruit ripening. In contrast, *PaNAC059* and *PaNAC094* show relatively low expression across all tissue types, suggesting a less prominent role in these stages. These findings emphasize the complex regulation of *PaNACs* in sweet cherry, in which specific genes reveal the role of key developmental processes such as bud formation, fruit development, and shoot growth.

### 2.7. Expression Analysis of Selected PaNAC Genes Under Abiotic Stress

**Figure 6:**
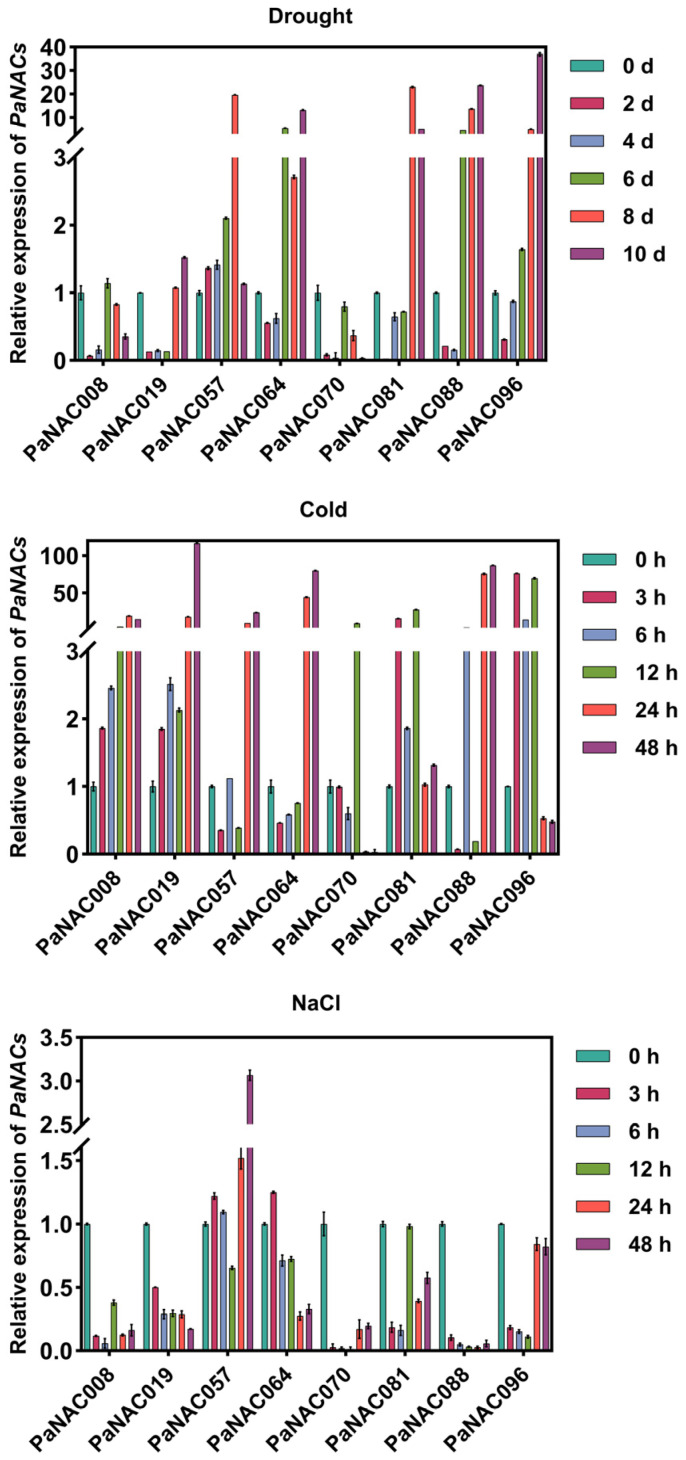
Expression patterns of selected *PaNAC* genes under different abiotic stresses (drought, cold, and NaCl). Gene expression levels were normalized to the *PaActin* reference gene as an internal control. Bars represent the mean ± SD of three biological replicates.

Numerous studies have shown that *NAC* genes play a crucial role in responding to abiotic stresses. In this study, we selected one gene from each of these six abiotic stress-related subfamilies, along with one gene from each of the two cherry-specific subfamilies, for expression analysis under drought, cold, and salt stress conditions [36]. The results revealed significant expression differences among these *PaNAC* genes under different stress (Figure 6). During drought treatment, *PaNAC057*, *PaNAC081*, *PaNAC088*, and *PaNAC096* significantly upregulated at later stages, suggesting their involvement in drought response (Figure 6). Under cold treatment, *PaNAC096* exhibited a sharp increase at 3–12 h, while *PaNAC057*, *PaNAC064* and *PaNAC088* were significantly upregulated at later stages (24 h and 48 h), indicating their roles in both early and prolonged cold stress responses (Figure 6). Under salt treatment, *PaNAC057* exhibits sustained upregulation during the 24–48 h period, suggesting its involvement in the late-stage response to salt stress (Figure 6). In contrast, *PaNAC070* and *PaNAC088* exhibited relatively low expression under salt stress conditions, suggesting their limited involvement in salt stress responses. In summary, *PaNAC* genes play a significant role in the response of sweet cherry to abiotic stresses, demonstrating functional diversity and specialized roles in stress adaptation.

## 3. Discussion

The *NAC* gene family is one of the largest and most extensively studied transcription factor families in plants, playing a crucial role in plant growth, development, and responses to abiotic stresses [18,36]. The genome-wide identification of *NAC* genes has been studied in multiple species, but little is known about this gene family in sweet cherry [35,37,38]. In this study, a total of 130 *PaNAC* genes were identified in sweet cherry, which is less than the 151, 142, 185, and 181 *NAC* genes identified in *Oryza sativa* [31], *Actinidia eriantha* [39], *Pyrus bretschneideri Rehder* [40], and *Musa acuminata* [41], but more than the 115, 93, and 79 *NAC* genes identified in *A. thaliana* [7], *Solanum lycopersicum* [35], and *Vitis vinifera* [34], respectively. This difference may be related to genetic variation and whole-genome duplication (WGD) events during plant evolution.

Similarly to the findings in rice [20], and Arabidopsis [7], the physicochemical properties of *PaNAC* genes vary widely and are unevenly distributed across chromosomes; however, their gene and protein structures are relatively conserved. Based on their homology to *NAC* genes in Arabidopsis, we classified these genes into 21 subfamilies. The phylogenetic tree revealed two sweet cherry-specific subfamilies, *Pa_NAC1* and *Pa_NAC2*. Interestingly, similar cases were also found in the *NAC* gene family of kiwifruit [39] and tomato [35]. Therefore, we speculate that these sweet cherry-specific *NAC* genes may have specialized functions in sweet cherry plants. Additionally, the absence of certain subfamilies in sweet cherry, such as *TIP* and *ANAC063*, indicates that these genes may have been lost after the divergence of sweet cherry and Arabidopsis, highlighting the distinct evolutionary paths of these species.

Structural differences in genes play a key role in the evolution of gene families, contributing to our understanding of genetic diversity and environmental adaptability in plants [42,43]. The gene structure analysis of *PaNAC* genes in sweet cherry reveals significant variability, with most genes containing at least one intron and a range of exons, highlighting the functional diversity within the *NAC* family. The absence of introns in some genes may indicate differences in their regulatory mechanisms and evolutionary origins. Motifs, as conserved sequences, are integral to performing specific biological functions, with each motif typically corresponding to a particular functional domain [44]. The presence of six conserved motifs was observed across the majority of PaNAC proteins, which was similar to the results of *Prunus mume* [45]. The variability in motif numbers, particularly in PaNAC120, PaNAC011, PaNAC054, and PaNAC109, implies that these genes may have specialized functions, which could be associated with their unique physiological roles in sweet cherry.

Duplication events are crucial to plant evolutionary patterns, with tandem and segmental duplications contributing to gene family expansion and genomic complexity [46]. Numerous studies have shown that gene family expansion primarily occurs through tandem, whole-genome, and segmental duplications [46,47]. In this study, we discovered that the *PaNAC* gene family expanded mainly through tandem and segmental duplication events, which is consistent with the findings in *P. mume* [45]. Additionally, *PaNAC* has undergone strong purifying selection during evolution, which may have contributed to the stability of its function [48,49].

Cis-acting elements in plant promoters are crucial for the regulation of gene expression, particularly in response to environmental signals and developmental cues [50,51]. We discovered that the promoters of most *PaNAC* genes contain multiple cis-acting elements responsive to hormones, such as ABRE, GARE-motif, and CGTCA-motif, which was similar to the results of apple [33]. This suggests that *PaNAC* genes play a role in regulating hormone signaling pathways, which may be related to their involvement in growth, development, and responses to abiotic stresses.

Extensive studies have shown that NAC transcription factors play a crucial role in regulating various physiological processes, including organ development, fruit maturation, and hormone signaling [52,53,54,55]. We analyzed publicly available transcriptome data, and the results indicated that *NAC048* and *PaNAC049* exhibit higher expression during fruit ripening, indicating that they likely play important regulatory roles in this stage. In contrast, *PaNAC059* and *PaNAC094* show relatively low expression during fruit ripening, suggesting a less prominent role in these stages. Furthermore, we found that among the four gene cis-acting elements, only *PaNAC048* and *PaNAC049* contain the ERE and ABRE cis-acting elements involved in fruit ripening. These may be factors contributing to the differential expression of *PaNAC048/PaNAC049* and *PaNAC059/PaNAC094*, prompting further investigation.

Ten and five *NAC* genes related to drought tolerance were identified in *Cicer arietinum* [56] and *Solanum tuberosum* [57], respectively. Additionally, 31 and 15 *NAC* genes related to cold tolerance were identified in *Dendrobium officinale* [58] and *P. smume* [45]. Ten *NAC* genes related to salt tolerance were identified in *Isatis indigotica* [59]. In wheat, the overexpression of *TaNAC071-A* significantly enhanced drought tolerance through improved water-use efficiency and the increased expression of stress-responsive genes [60]. In rice, OsNAC5 enhances the stability of OsABI5, thereby regulating the expression of cold-responsive (*COR*) genes and fine-tuning plant responses to cold stress [25]. In soybean, the transgenic overexpression of *GmNTL1* in soybean increases the H_2_O_2_ levels and K^+^/Na^+^ ratio in the cell, promotes salt tolerance, and increases yield under salt stress [61]. This suggests that the NAC TF family is involved in plant responses to various abiotic stresses.

‘Gisela 6’ (G6) is widely used as a rootstock in commercial sweet cherry production due to its strong compatibility and adaptability with *P. avium* scions. In our study, analyzing the expression of *PaNAC* genes under abiotic stress conditions in G6 provides valuable insights into rootstock-mediated stress responses. G6 is a hybrid derived from *P. cerasus* (sour cherry) and *P. canescens*, and notably, *P. cerasus* itself originated from a natural hybridization between *P. avium* and *P. fruticose* [62,63], which suggests that, from a phylogenetic perspective, G6 shares a certain level of genetic relatedness with *P. avium*. Moreover, our sequence alignment results revealed a high degree of sequence similarity between *NAC* genes from sweet cherry and G6 (Table S4). We analyzed the expression of *PaNAC* genes under abiotic stress in G6. The results showed that *PaNAC057/PaNAC081/PaNAC088/PaNAC096*, *PaNAC057/PaNAC064/PaNAC088* and *PaNAC057* were significantly upregulated under drought stress, cold stress, and salt stress, respectively. Notably, *PaNAC057* is significantly induced under drought, cold, and salt stress treatments, suggesting that this gene may serve as a central hub in the coordinated response to multiple stresses. Nevertheless, more genetic evidence is required for further understanding the functions of *NAC* genes in response to abiotic stresses in sweet cherry.

## 4. Materials and Methods

### 4.1. Identification of NAC Genes in the Prunus avium

The sweet cherry (*P. avium*) v2.0 was obtained from the Rosaceae genome database (GDR, https://www.rosaceae.org/ (accessed on 21 June 2024)). The Arabidopsis thaliana *NAC* gene sequences were obtained from the Arabidopsis Information Resource (TAIR, https://www.arabidopsis.org/ (accessed on 21 June 2024)). To identify all the *NAC* gene family members in sweet cherry, we utilized the HMMER 3.3.2 Hidden Markov Model (PF02365) to screen candidate proteins with E-values below 1 × 10^−10^ and subsequently constructed a species-specific Hidden Markov Model for sweet cherry. We also used the BLASTP program to search against all genome protein sequences of sweet cherry, using Arabidopsis thaliana *NAC* gene sequences with an E-value < 1 × 10^−5^ as queries. The results of the secondary search and BLASTP were further confirmed using SMART (http://smart.embl-heidelberg.de/ (accessed on 23 June 2024)), PFAM (http://pfam.xfam.org (accessed on 23 June 2024)), and NCBI-CDD (https://www.ncbi.nlm.nih.gov/cdd/ (accessed on 23 June 2024)) to ensure reliability. The physicochemical properties of *PaNAC* genes, including amino acid composition, molecular weight (MW), and theoretical isoelectric point, were analyzed using the Expasy server (www.expasy.org/ (accessed on 26 June 2024)). Subcellular localization was predicted with the online tool, Cell-Ploc-2.0 (http://www.csbio.sjtu.edu.cn/bioinf/Cell-PLoc-2/ (accessed on 26 June 2024)).

### 4.2. Chromosomal Mapping, Duplication and Syntenic Analysis of PaNAC Genes

The positions of *PaNAC* genes and the size of each chromosome were extracted from the GFF3 annotation files in the sweet cherry library, and the *PaNAC* genes were mapped to sweet cherry chromosomes using MapChart v2.32 software [64]. Duplication events and the synteny analysis of *PaNAC* genes were performed using MCScanX software [65] with default parameters, and Circos v0.69 [66] was used to visualize the synteny relationships of the *PaNAC* genes.

### 4.3. Phylogenetic Analysis and Classification of PaNAC Genes

The full-length protein sequences of PaNACs and AtNACs were aligned using ClustalW in MEGA X [67]. A phylogenetic tree was then constructed in MEGA X with the Poisson model, employing the NJ method with pairwise gap deletion and 1000 bootstrap iterations. Based on the AtNAC classification, the PaNACs were categorized into distinct subfamilies.

### 4.4. Gene Structure and Motif Analysis of PaNAC Genes

Conserved motifs in PaNAC proteins were identified using the MEME tool with the following parameters: a maximum of 10 motifs and motif widths ranging from 6 to 50 residues [68]. The gene structure of *PaNACs* was visualized through the Gene Structure Display Server (GSDS2.0, https://gsds.gao-lab.org/index.php (accessed on 8 July 2024)).

### 4.5. Promoter Analysis of PaNAC Genes

The 1500 bp upstream sequences of the *PaNAC* genes start codon (ATG) were extracted from the sweet cherry genome using a Pythonscript (Python v3.7.10) and analyzed for cis-acting elements with PlantCARE (accessed on 15 July 2024) [69]. The distribution of these elements on each promoter was visualized using GSDS2.0.

### 4.6. Gene Expression Analysis of Sweet Cherry Tissues and Developmental Stages

The transcriptional patterns of *PaNAC* genes across various tissues were obtained from RNA sequencing data for sweet cherry in the DDBJ/EMBL/GenBank Sequence Read Archive (SRA) databases (codes SUB7211514). Clean reads were aligned to the *P. avium* genome using Hisat2 v2.1.0 [70] with default settings. FeatureCounts v2.0.0 [71] and StringTie [72] were then employed to quantify gene expression levels in transcripts per million (TPM). The TPM values of the *PaNAC* genes were transformed by log2(TPM + 1). Finally, heat maps of *PaNAC* genes were generated using the pheatmap function in the R package (pheatmap, https://cran.r-project.org/web/packages/pheatmap/index.html (accessed on 2 August 2024)).

### 4.7. Plant Materials and Stress Treatments

Sequence alignment revealed high homology (98.68–100%) between the selected *PaNAC* genes in *P. avium* and their orthologs in ‘Gisela 6’, which was subsequently used for tissue culture experiments (Table S4). The ‘Gisela 6’ tissue culture seedlings were planted in a growing medium containing Murashige and Skoog (MS) medium, 30 g L^−1^ agar, 0.5 mg L^−1^ indole-3-butyric acid (IBA), 0.5 mg L^−1^ 3-indoleacetic acid (IAA), and 0.5 g L^−1^ activated carbon. After 30 days of growth, the seedlings were transplanted into a substrate for further cultivation. Uniformly developed plants were selected for subsequent treatments. Following full hydration, watering was ceased, and leaves were sampled at 0, 2, 4, 6, 8, and 10 days post-treatment. For cold stress, plants were maintained at 4 °C under a 16 h light/8 h dark cycle, and leaf samples were collected at 0, 3, 6, 12, 24, and 48 h post-treatment. For salt stress, the plants were treated with 200 mM NaCl via root irrigation, and leaf samples were taken at 0, 3, 6, 12, 24, and 48 h post-treatment. All collected samples were immediately frozen in liquid nitrogen and stored at −80 °C. Each treatment included at least three biological replicates.

### 4.8. RNA Extraction and qRT-PCR

Total RNA was isolated using the Plant Total RNA Isolation Kit Plus (Foregene, Chengdu, China), and reverse transcription was carried out using the Hifair III 1st Strand cDNA Synthesis Kit (Yeasen, Shanghai, China). Specific primers for *PaNAC* were designed using Primer 5.0 software and are listed in Supplementary Table S3. qRT-PCR was performed using the SYBR Green Premix Pro Taq HS qPCR Kit (Accurate Biotechnology, Changsha, China) on a QuantStudio 3 Real-Time PCR System (Life Technologies, Carlsbad, CA, USA). The reaction procedure’s settings were as follows: 95 °C for 30 s, 40 cycles of 95 °C for 5 s, and 60 °C for 30 s. For the rest of the parameters, default settings were used. Each sample and reaction had at least three biological replicates and each reaction was performed with three technical replicates, respectively. The relative expression levels of *PaNAC* were calculated using the 2^−ΔΔCt method [73], with the actin gene of sweet cherry serving as the reference gene.

## 5. Conclusions

In this study, we identified 130 *PaNAC* genes in sweet cherry and classified them into 21 subfamilies based on phylogenetic analysis. Two sweet cherry-specific subfamilies, *Pa_NAC1* and *Pa_NAC2*, were identified, suggesting species-specific functions. Gene structure and motif analysis revealed high conservation, while variations indicated functional diversification. Duplication and synteny analyses showed that gene expansion was primarily driven by segmental and tandem duplications, with purifying selection shaping *PaNAC* gene evolution. Cis-element analysis suggested their involvement in hormone signaling and stress responses. Tissue-specific expression patterns and qRT-PCR validation further confirmed their roles in plant development and abiotic stress adaptation. These findings provide a foundation for future research on the regulatory mechanisms of *PaNAC* genes in growth, development, and stress tolerance.

## Data Availability

All experimental data are provided in the main text and/or Supplementary Materials.

## Supplementary Materials

The following supporting information can be downloaded at https://www.mdpi.com/article/10.3390/plants14081201/s1. Figure S1: Distribution of *PaNAC* genes on *P. avium* chromosomes; Figure S2: The cis-element composition in the promoter regions of *PaNAC* genes; Table S1: Predicted physicochemical properties of 130 NAC proteins; Table S2: *Ks*, *Ka*, and *Ka/Ks* analysis of duplicated *NAC* gene pairs; Table S3: Primer sequences for qRT-PCR analysis of *PaNAC* gene expression. Table S4 Sequence homology of eight *P. avium* ‘Tieton’ genes in the hybrid rootstock *P. canescens* × *P. cerasus* ‘Gisela 6’.
