# Supplementary material for: Genome-Wide Identification, Characterization, and Expression Analysis of the NAC Transcription Factor Family in Sweet Cherry (Prunus avium L.)
Source: Plants (Basel). 2025 Apr 12;14(8):1201. doi: 10.3390/plants14081201 (PMC12030308; doi:10.3390/plants14081201)
Supplement: Supplementary file 1 [file plants-14-01201-s001.zip › Figures S1 and S2.pdf]

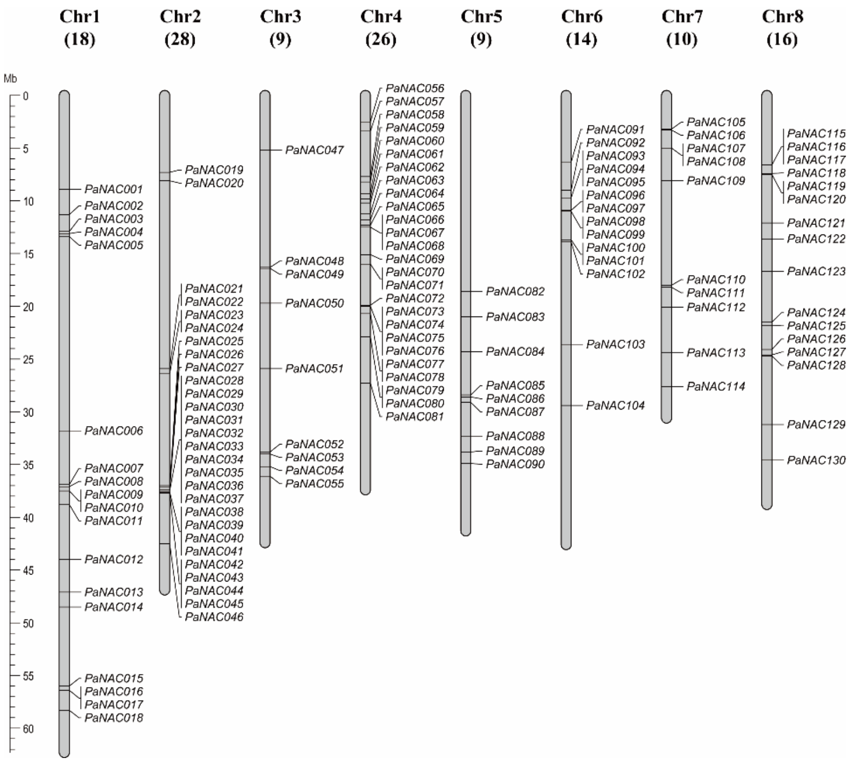

Figure S1. Distribution of PaNAC genes on *P. avium* chromosomes

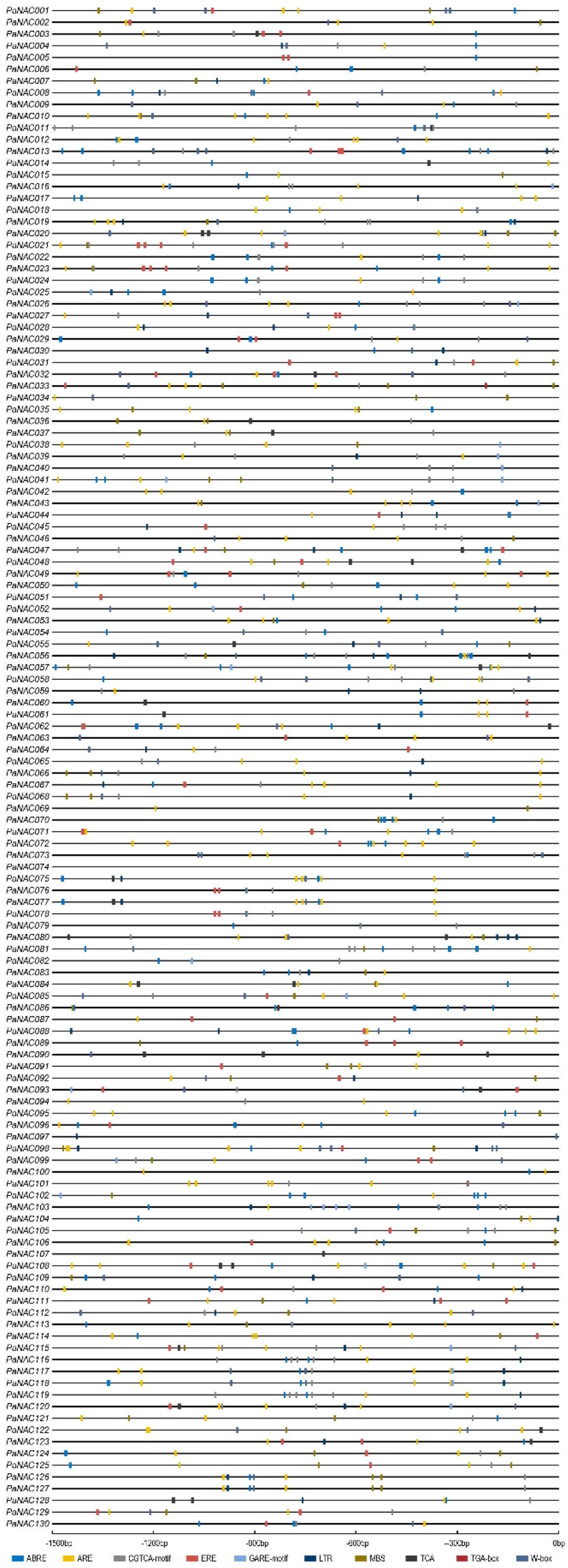

Figure S2. The cis-element composition in the promoter regions of *PaNAC* genes.
